# Network pharmacology- and molecular docking-based analyses of the antihypertensive mechanism of *Ilex kudingcha*

**DOI:** 10.3389/fendo.2023.1216086

**Published:** 2023-08-17

**Authors:** Fei Liao, Muhammad Yousif, Ruya Huang, Yanlong Qiao, Yanchun Hu

**Affiliations:** ^1^ Key Laboratory of Animal Disease and Human Health of Sichuan Province, College of Veterinary Medicine, Sichuan Agricultural University, Wenjiang, China; ^2^ Department of Animal Husbandry and Fisheries, Guizhou Vocational College of Agriculture, Qingzhen, China

**Keywords:** *Ilex kudingcha*, hypertension, network pharmacology, molecular target, molecular dynamic simulations

## Abstract

Herein, network pharmacology was used to identify the active components in *Ilex kudingcha* and common hypertension-related targets. Gene Ontology (GO) and Kyoto Encyclopedia of Genes and Genomes (KEGG) enrichment analyses were conducted, and molecular docking was performed to verify molecular dynamic simulations. Six active components in *Ilex kudingcha* were identified; furthermore, 123 target genes common to hypertension were identified. Topological analysis revealed the strongly associated proteins, with *RELA*, *AKT1*, *JUN*, *TP53*, *TNF*, and *MAPK1* being the predicted targets of the studied traditional Chinese medicine. In addition, GO enrichment analysis revealed significant enrichment of biological processes such as oxidative stress, epithelial cell proliferation, cellular response to chemical stress, response to xenobiotic stimulus, and wound healing. Furthermore, KEGG enrichment analysis revealed that the genes were particularly enriched in lipid and atherosclerosis, fluid shear stress and atherosclerosis, and other pathways. Molecular docking revealed that the key components in *Ilex kudingcha* exhibited good binding potential to the target genes *RELA*, *AKT1*, *JUN*, *TP53*, *TNF*, and *IL-6*. Our study results suggest that *Ilex kudingcha* plays a role in hypertension treatment by exerting hypolipidemic, anti-inflammatory, and antioxidant effects and inhibiting the transcription of atherosclerosis-related genes.

## 1 Introduction

Hypertension is the primary risk factor for mortality and the most frequent reason for renal, cardiovascular, and cerebrovascular disorders. Globally, approximately 1 billion individuals suffer from hypertension; it is directly responsible for 13% of all fatalities (1). In China, one in four individuals suffers from hypertension, with 40% suffering from severe hypertension. However, most individuals are unaware of their disease and receive inadequate care (2). The use of antihypertensive drugs is the most effective method for lowering blood pressure and preventing cardiovascular events; however, only 32.5% of patients with hypertension worldwide have controlled blood pressure (3). In general, blood pressure is controlled by using a combination of two or more antihypertensive drugs (4). In less developed areas, maintaining adequate blood pressure control is difficult because the medication is lowered (5). Epidemiological studies have reported that the prevalence of resistant hypertension is 10% among individuals with hypertension, with high cardiovascular risk in this patient cohort (1). Furthermore, despite conscientious clinical management, many adults with resistant hypertension fail to achieve their recommended blood pressure treatment targets on three antihypertensive medications or require more than four medications to achieve their targets (6).

Considering the significant role of diet in blood pressure homeostasis, the High Blood Pressure Clinical Practice Guidelines of the American College of Cardiology/American Heart Association recommend dietary strategies as a practical and acceptable approach to control blood pressure (7). Hippocrates mentioned that “Let food be thy medicine and medicine be thy food” (8). In China, *Ilex* species exhibit a broad geographic range, and some species have been used to develop daily herbal tea blends. *Ilex kudingcha*, a Chinese herbal tea, possesses many health-improving properties (9). It is a well-known traditional Chinese beverage in Southeast Asia. *Ilex kudingcha* contains saponins, polyphenols, and flavones and exerts anti-inflammatory (10), antioxidative (11), antiaging (12), anticancer (13, 14), antiobesity (15, 16), antihypertensive, and antidiabetic effects (17).

However, the ingredients and molecular mechanisms underlying its antihypertensive effects remain unelucidated. Network pharmacology combines system network analysis with pharmacology, bioinformatics, and other disciplines to demonstrate the multicomponent and multitarget drug treatment process from the perspective of genes (18). By building a network associated with “disease–phenotype–gene–drug,” the distribution, molecular function, and signaling pathways of traditional Chinese medicine (TCM) compounds may be investigated. At present, the basis and mechanism of the pharmacodynamic components in TCMs are frequently predicted using network pharmacology approaches (19). Therefore, the present study aimed to use network pharmacology and molecular docking to determine the molecular targets and processes involved in hypotension treatment.

## 2 Materials and methods

### 2.1 Screening of active ingredients and target genes

The parameters oral bioavailability (OB) of 30% and drug-like (DL) characteristic of 0.18 p were used in the Traditional Chinese Medicine Systems Pharmacology (TCMSP) platform (http://lsp.nwu.edu.cn/tcmsp.php) to screen the active ingredients in *Ilex kudingcha*. The DrugBank database (https://www.drugbank.ca/) and peer-reviewed literature were used to identify the expected targets of the tested compounds. Furthermore, UniProt (https://www.uniprot.org/) was used to standardize gene names and compare target information. The *Ilex kudingcha*–ingredient–target regulatory network was developed using Cytoscape 3.9.1 software.

### 2.2 Collection of hypertension-related targets

The hypertension-related targets with high relevance were chosen using GeneCards (https://genecards.org). The targets with relevance scores of ≥1 were chosen based on the keyword “hypertension.” The hypertension-related targets were identified after weight removal and consolidation.

### 2.3 Identification of intersection targets and development of the “drug–disease–target” regulatory network

The hypertension-related target genes and intersection genes of *Ilex kudingcha* were acquired using R software. Then, the *Ilex kudingcha*–disease–target regulatory network was developed using Cytoscape 3.9.1 software.

### 2.4 Protein–protein interaction network and topological analysis

The intersection targets of *Ilex kudingcha* and hypertension were imported into the STRING database (https://string-db.org/cgi/input.pl). The species was limited to humans and a minimum interaction score of 0.9 was used. The key targets were imported into the Cytoscape program. The interaction network diagram of the target proteins of the active ingredients in *Ilex kudingcha* and hypertension-related target proteins was developed by sorting based on degree value.

### 2.5 Gene Ontology and Kyoto Encyclopedia of Genes and Genomes enrichment analyses

R 4.2.2 with the “colorspace,” “stringi,” and “ggplot2” packages was installed. The “DOSE,” “clusterProfiler,” and “annotationHub” Bioconductor packages were used for Gene Ontology (GO) and Kyoto Encyclopedia of Genes and Genomes (KEGG) enrichment analyses. For GO enrichment analysis, the function “enrichGO” was employed. For KEGG enrichment analysis, the database org.Hs.eg.db and the “enrich-KEGG” tool were used. The KEGG database (https://www.kegg.jp/) was also used (doi: 10.18129/ http://b9.bioc.org.Hs.eg.db). For the parameters of the two functions, species was set to “has,” and the filter values (*P*- and *q*-values) were set to 0.05. A bubble graph was prepared to visualize the top 10 enrichment findings, and Cytoscape 3.9.1 was used to develop the KEGG regulatory network.

### 2.6 Molecular docking

Three-dimensional (3D) protein conformations with a crystal resolution of <3, as determined using X-ray crystal diffraction, were gathered by searching the Protein Data Bank (PDB) (https://www.rcsb.org) for the target genes implicated in the first eight protein–protein interaction (PPI) findings. The primary active ingredients in *Ilex kudingcha* were retrieved in a two-dimensional (2D) structure format from the PubChem website. The sdf files were converted into the pdb file format using Discovery Studio 2019 software. PyMOL 4.3.0 software was used to separate the original ligand and protein structures and to dehydrate and remove the organics. Furthermore, AutoDock Vina 1.1.2 software was used to process the proteins as follows: non-polar hydrogen was added, the Gasteiger charge was calculated, the AD4 type was assigned, and the flexible bonds of small molecules/ligands were set to be rotatable. Based on the original ligand coordinates, the docking box was adjusted to include all protein structures. Furthermore, the receptor protein was set to a semiflexible butt joint, and the Lamarckian genetic algorithm was selected. The docking results were obtained by running autogrid4 and autodock4; as a result, the binding energies were revealed. PyMOL 4.3.0 and Discovery Studio 2019 software were used to analyze and visualize the forces in 3D and 2D.

### 2.7 Molecular dynamic simulation

To further elucidate the stability of protein–ligand binding, GROMACS 2023.36 software was used for molecular dynamic (MD) simulation. The AMBER force field was used to describe the proteins and ligand molecules. The water model was set to the SPC water model. The system temperature was set to a vacuum environment of 300 K, and the simulation time was 100 ns. First, the energy balance of the simulated system was determined by using the steepest gradient algorithm under the absolute vacuum environment. After energy balance, MD simulation was completed; thereafter, root mean square deviation (RMSD) analysis of the simulation results was conducted to analyze the relative binding stability between the two chains in the simulation process. Furthermore, root mean square fluctuation (RMSF), which demonstrates the structural adaptability of each protein residue, was used to analyze the flexibility and intensity of movement of the amino acid residues of the protein throughout the simulation. The radius of gyration (Rg) was used to characterize the compactness of the protein structure and changes in the looseness of the peptide chain during the simulation. VMD software was used to analyze the changes in the number of hydrogen bonds formed between the two chains over time as well as to observe whether the bond was stable from the interaction point of view.

## 3 Results

### 3.1 Screening of active ingredients and target genes

**Figure 1:**
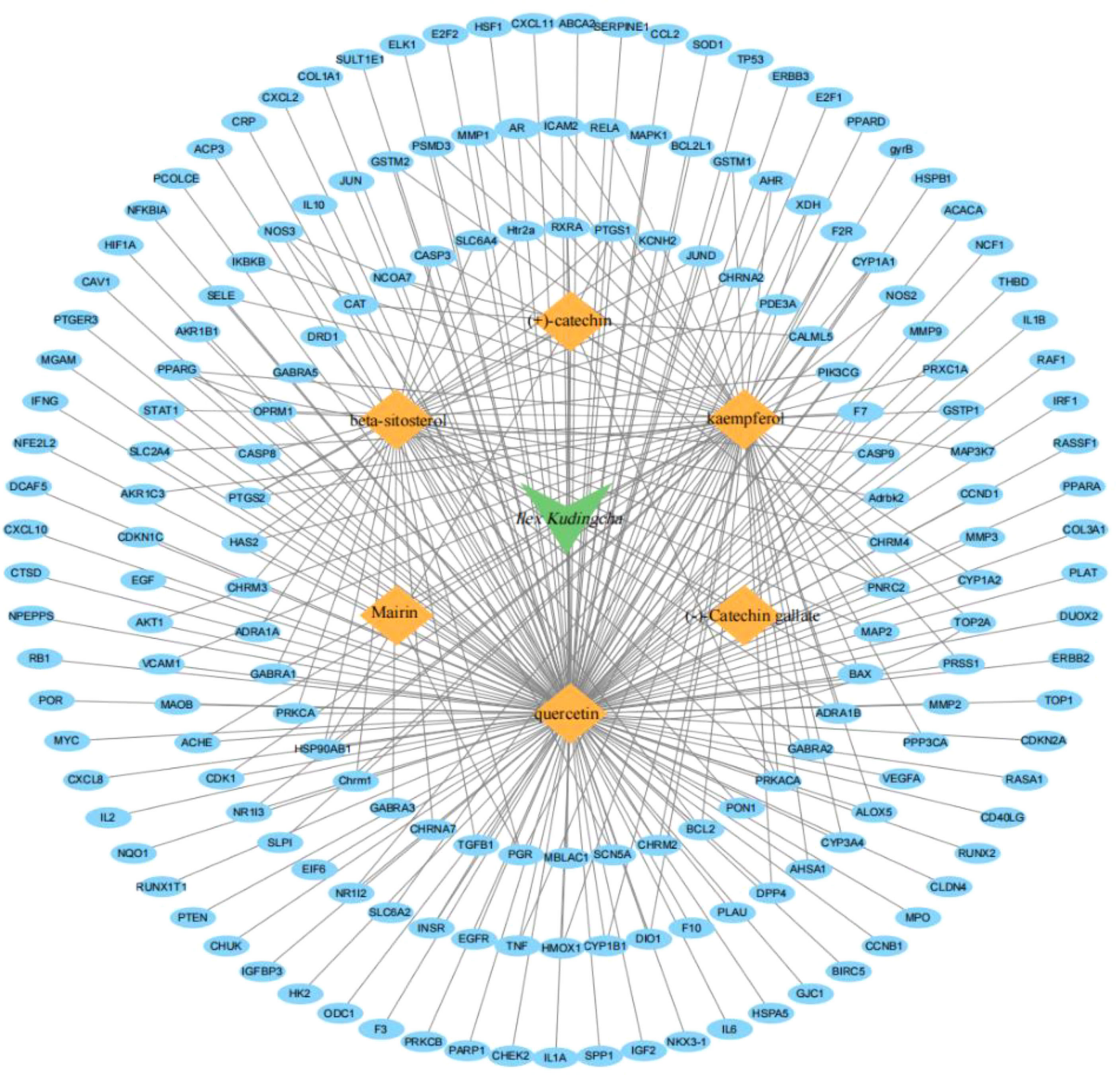
An illustration of the *Ilex kudingcha*–ingredient–target regulatory network.

The TCMSP database contains 94 active ingredients in *Ilex kudingcha*. They were screened using an OB of ≥30% and a DL of 0.18. Subsequently, six active ingredients were obtained (**Table 1**). The targets were predicted using DrugBank and UniProt. Finally, 179 targets were obtained (153 for quercetin, 1 for mairin, 63 for kaempferol, 37 for beta-sitosterol, 11 for (+)-catechin, and 1 for (−)-catechin gallate). **Figure 1** illustrates the *Ilex kudingcha*–ingredient–target regulatory network.

**Table 1:** The active ingredients of *Ilex kudingcha*.

Herbs	ID	Compound	OB (%)	DL
*Ilex kudingcha*	MOL000098	Quercetin	46.43	0.28
*Ilex kudingcha*	MOL000211	Mairin	55.38	0.78
*Ilex kudingcha*	MOL000422	Kaempferol	41.88	0.24
*Ilex kudingcha*	MOL000358	Beta-sitosterol	36.91	0.75
*Ilex kudingcha*	MOL000492	(+)-Catechin	54.83	0.24
*Ilex kudingcha*	MOL006504	(−)-Catechin gallate	53.57	0.75

### 3.2 Identification of intersection targets and development of the “drug–disease–target” regulatory network

**Figure 2:**
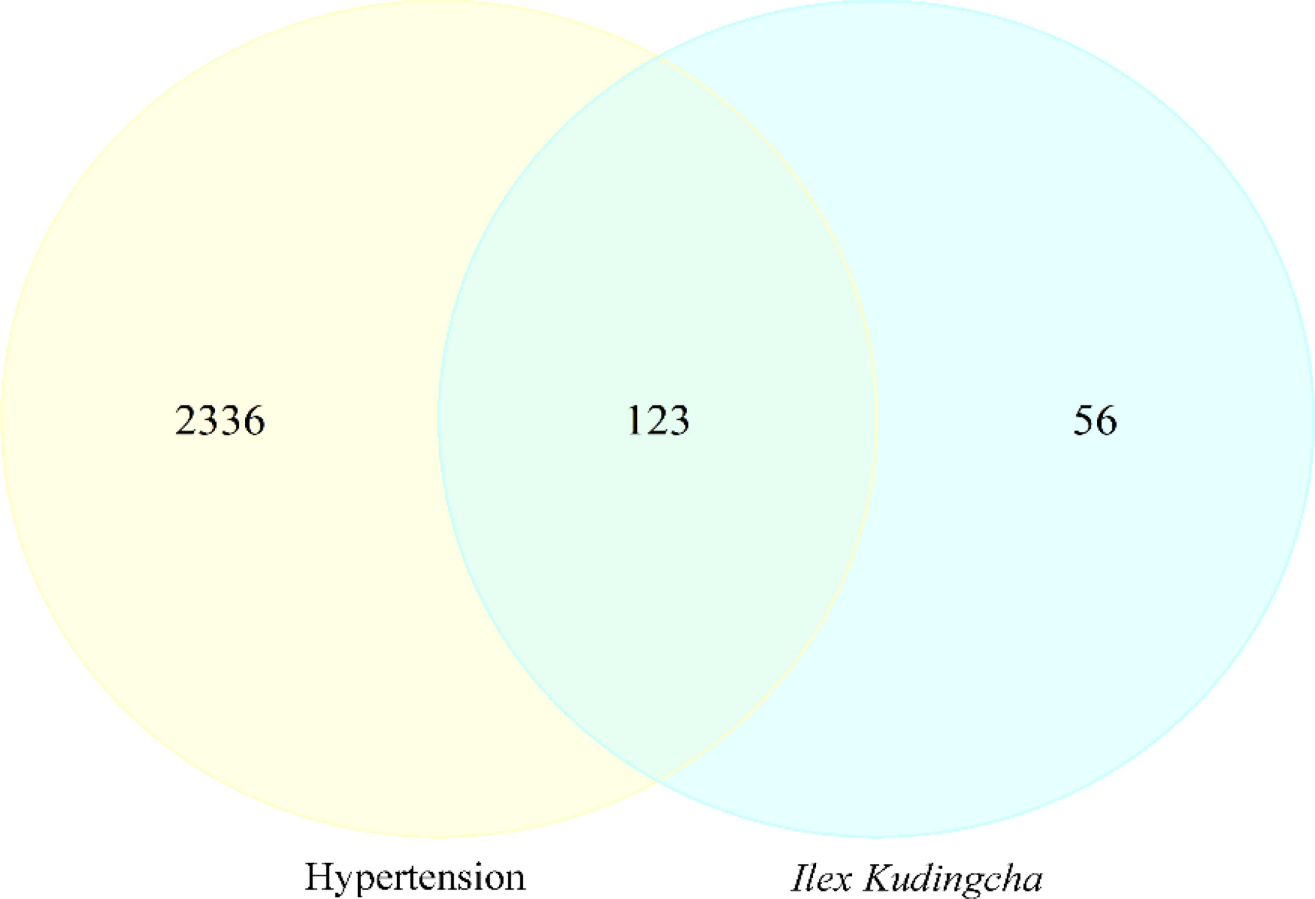
A Venn diagram of the *Ilex kudingcha–*hypertensive target.

**Figure 3:**
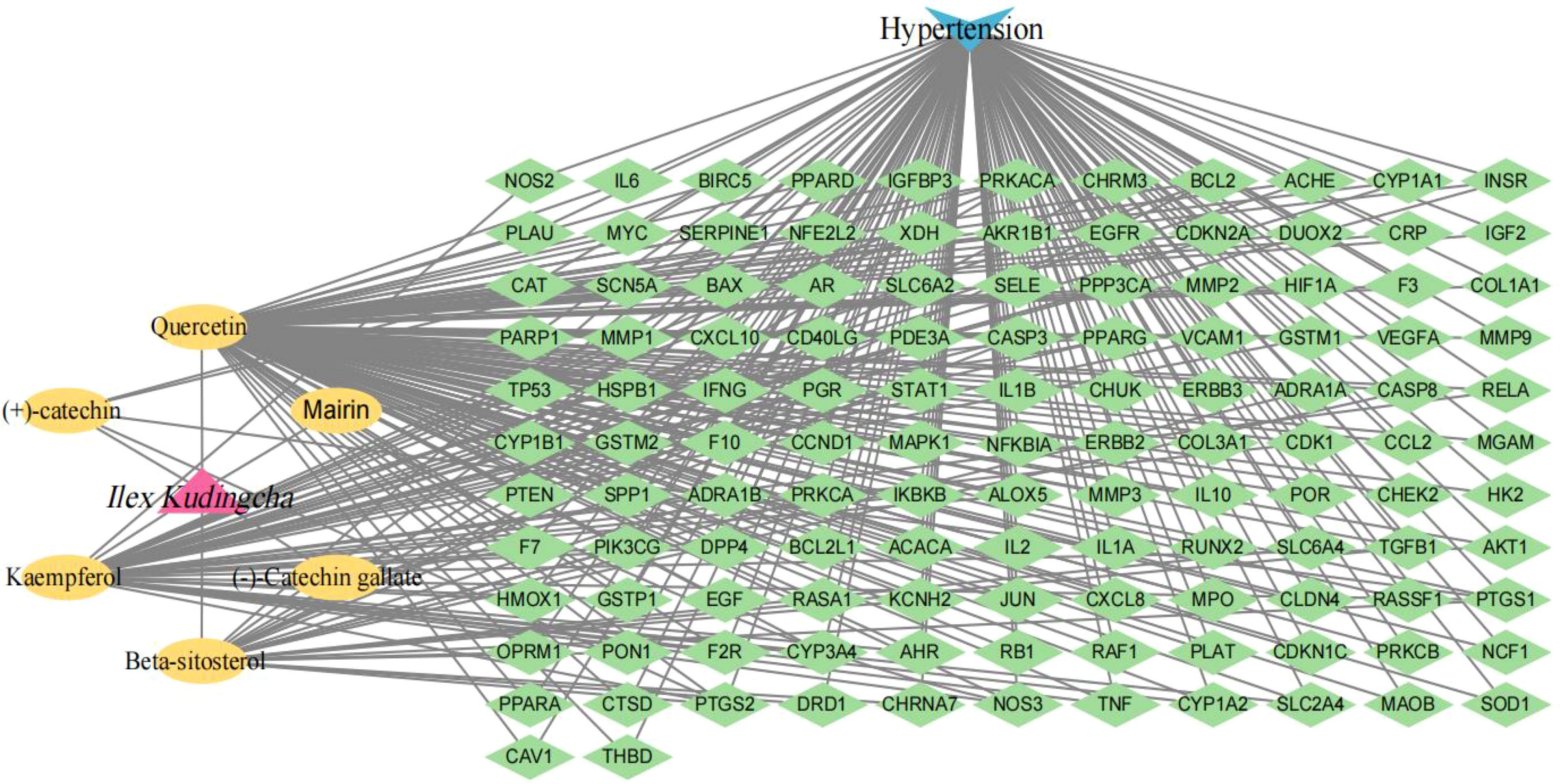
An illustration of the *Ilex kudingcha–*ingredient–target–hypertension regulatory network.

A total of 2,459 hypertension-related target genes were obtained, with 123 intersection targets between *Ilex kudingcha* and hypertension (**Figure 2**). **Figure 3** illustrates the *Ilex kudingcha*–ingredient–target–hypertension regulatory network. The active ingredients kaempferol and quercetin were associated with 42 and 111 target genes, respectively. Therefore, they were classified as multitarget and multieffect compounds. The genes *PTGS1*, *PTGS2*, *PRKACA*, and *PPARG* were associated with the highest number of active components.

### 3.3 PPI network and topological analyses

**Figure 4:**
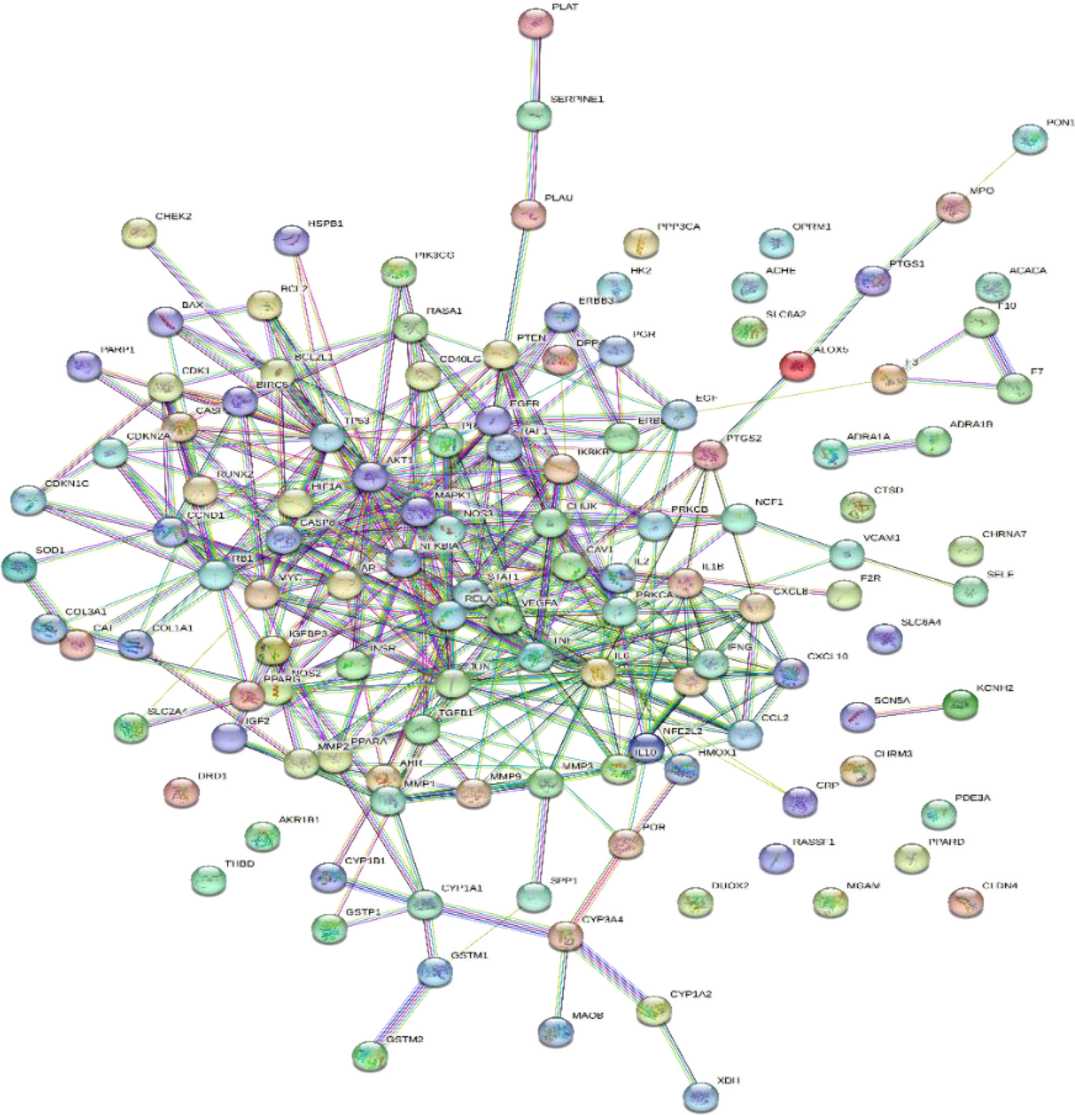
The protein–protein interaction network.

**Figure 5:**
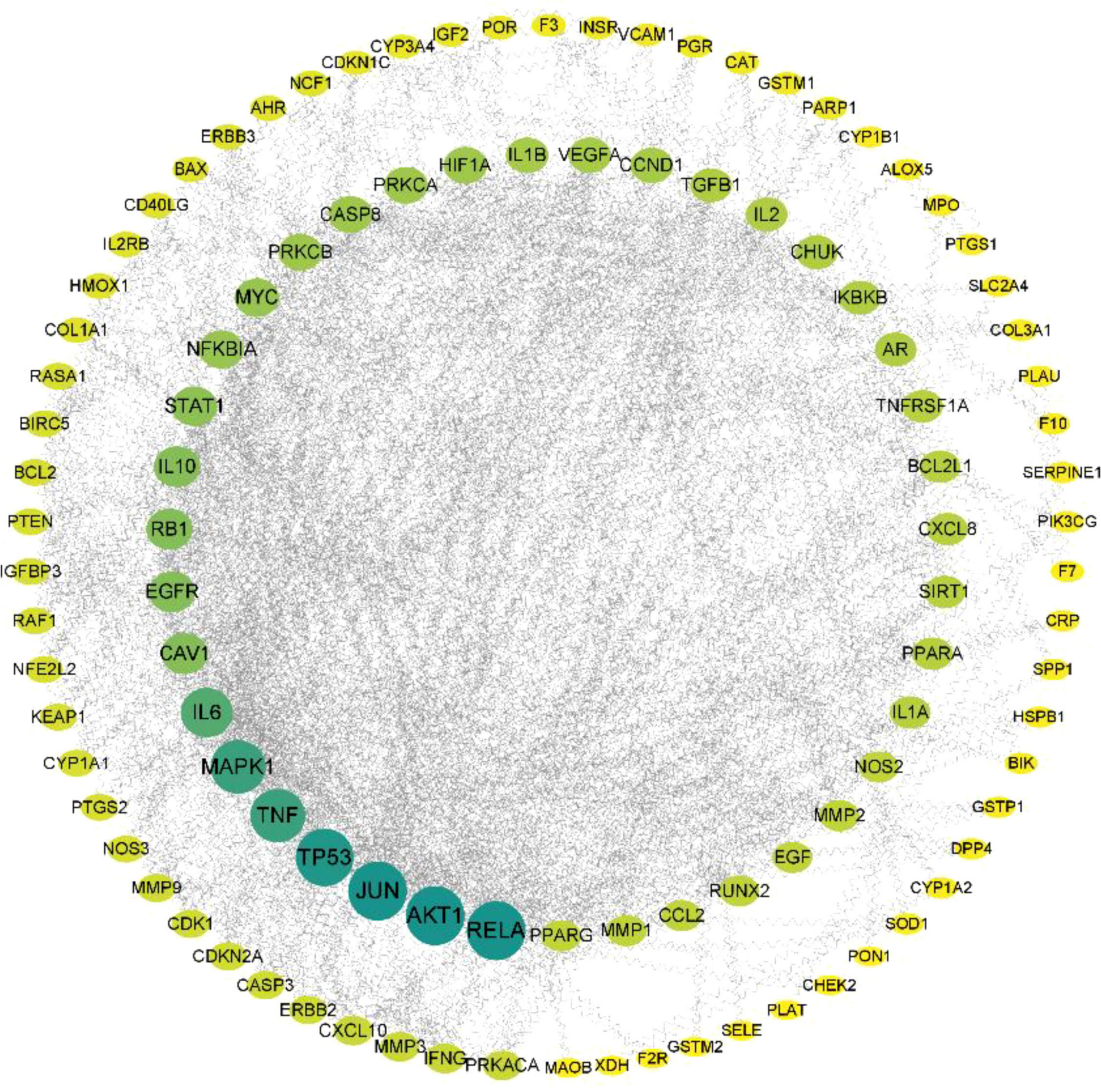
An illustration of the core target sequencing.

The 123 intersection targets were imported into the STRING database to construct the PPI network; the species was limited to humans and a minimum interaction score of 0.9 was used. As shown in **Figure 4**, 123 protein nodes and 377 edges were obtained for the intersection genes. In the PPI network, the degree centrality of a node is simply the number of edges it has. The higher the degree, the more central the node is. The interaction network diagram of the target proteins of the active ingredients and hypertension-related target proteins of *Ilex kudingcha* was obtained by ranking the proteins based on their degree value. Finally, 105 core target genes were obtained. As demonstrated in **Figure 5**, the higher the degree value, the darker the color and the larger the circle. The main targets were *RELA*, *AKT1*, *JUN*, *TP53*, *TNF*, and *MAPK1*, with degree values of 58, 58, 58, 56, 50, and 50, respectively.

### 3.4 Analysis of GO function and KEGG enrichment of related targets

**Figure 6:**
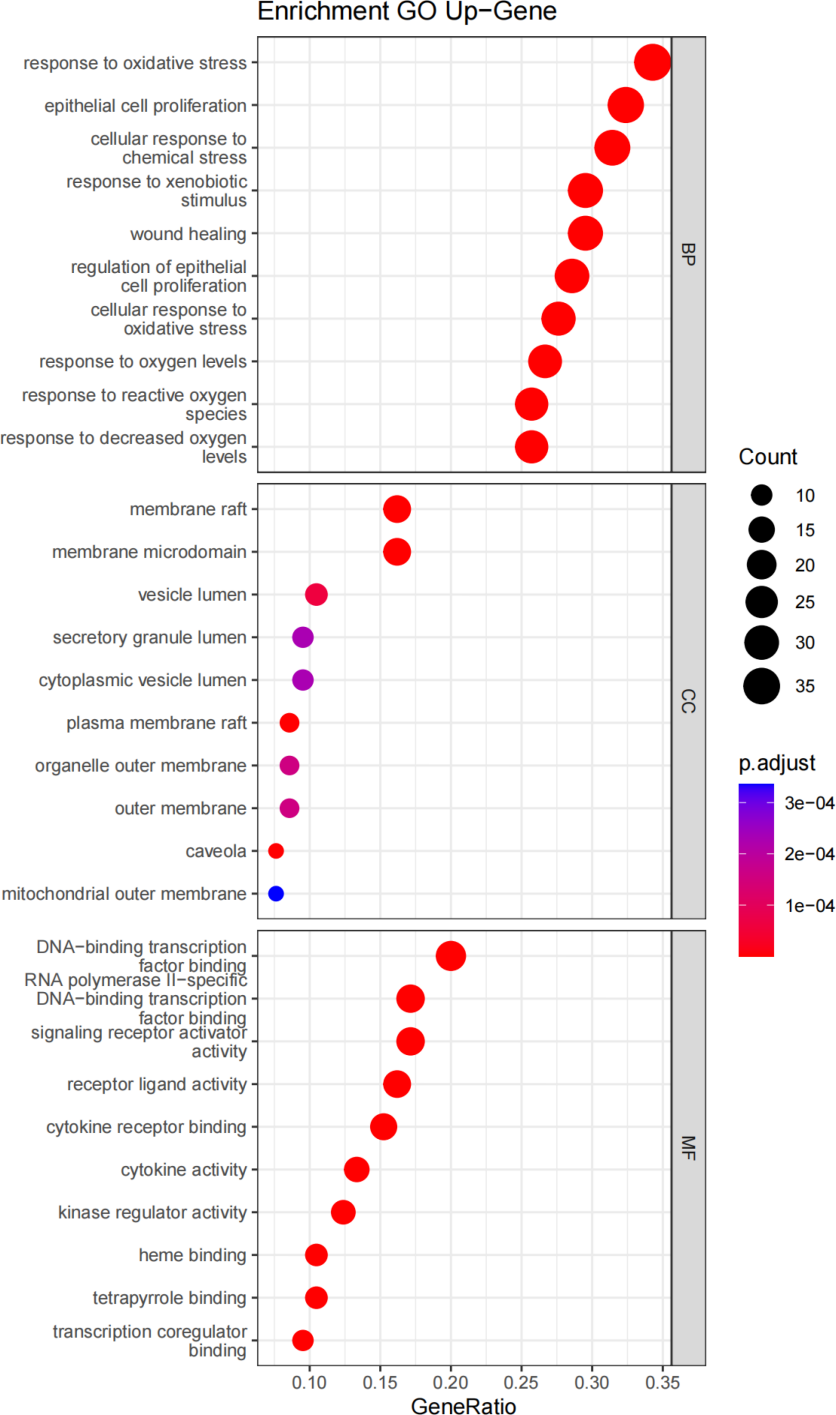
GO enrichment analysis of *Ilex kudingcha* targets in hypertension treatment. The horizontal axis of the BP, CC, and MF bubble diagram indicates the number of genes enriched in each item, while the color represents the enrichment significance based on the corrected *P*-value.

GO enrichment analysis revealed the gene functions at three levels: biological process (BP), cellular component (CC), and molecular function (MF). BP was mainly associated with response to oxidative stress, epithelial cell proliferation, cellular response to chemical stress, response to xenobiotic stimulus, and wound healing. CC was mainly associated with the membrane raft, membrane microdomain, vesicle lumen, secretory granule lumen, cytoplasmic vesicle lumen, and plasma membrane raft. MF was mainly associated with DNA-binding transcription factor binding, RNA polymerase II-specific DNA-binding transcription factor binding, signaling receptor activator activity, receptor–ligand activity, cytokine receptor binding, and cytokine activity (**Figure 6**).

**Figure 7:**
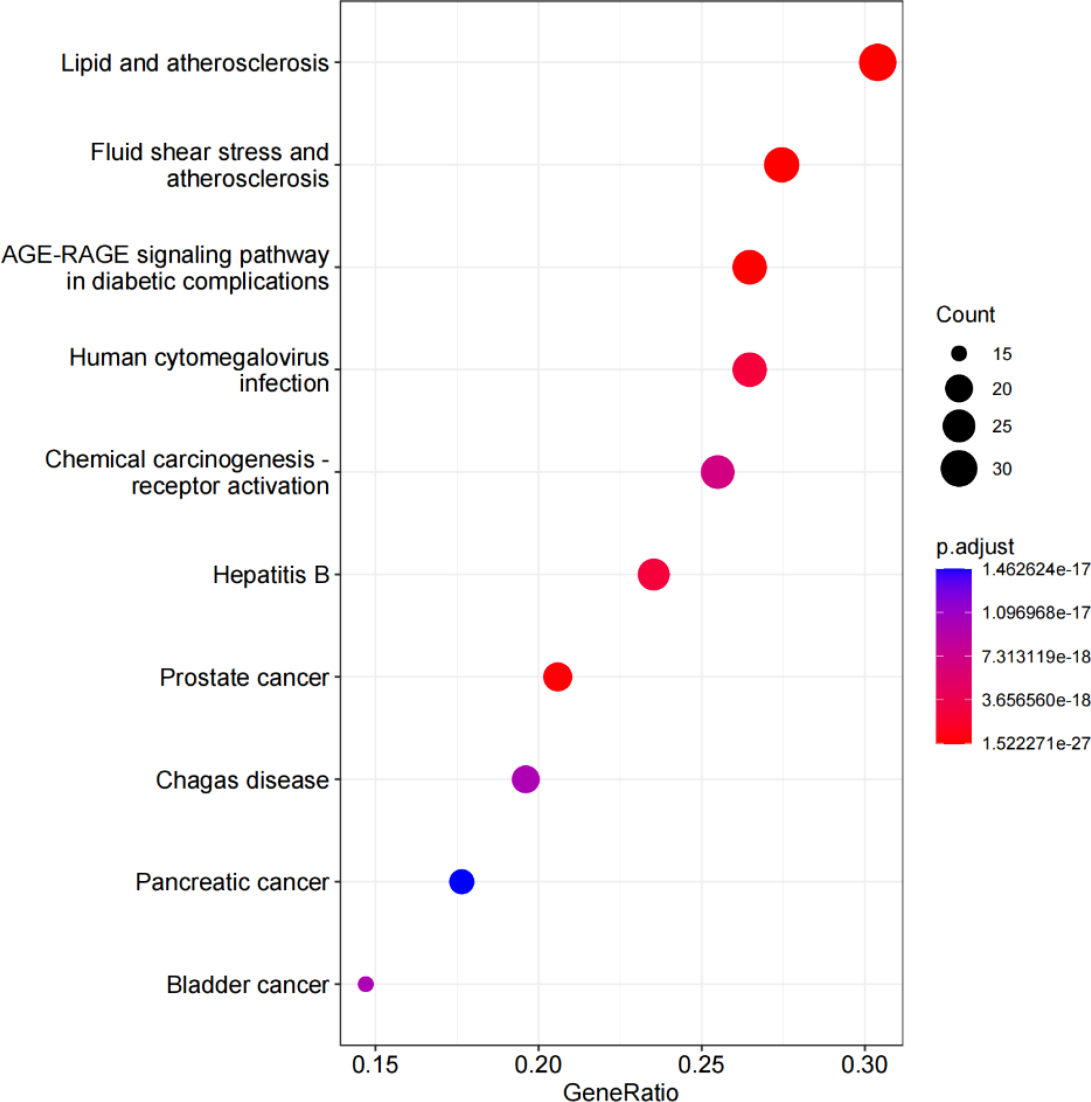
The KEGG bubble. The horizontal axis of the KEGG bubble diagram represents the gene proportion enriched in each entry, while the vertical axis indicates the enrichment degree according to the corrected *P*-value.

**Figure 8:**
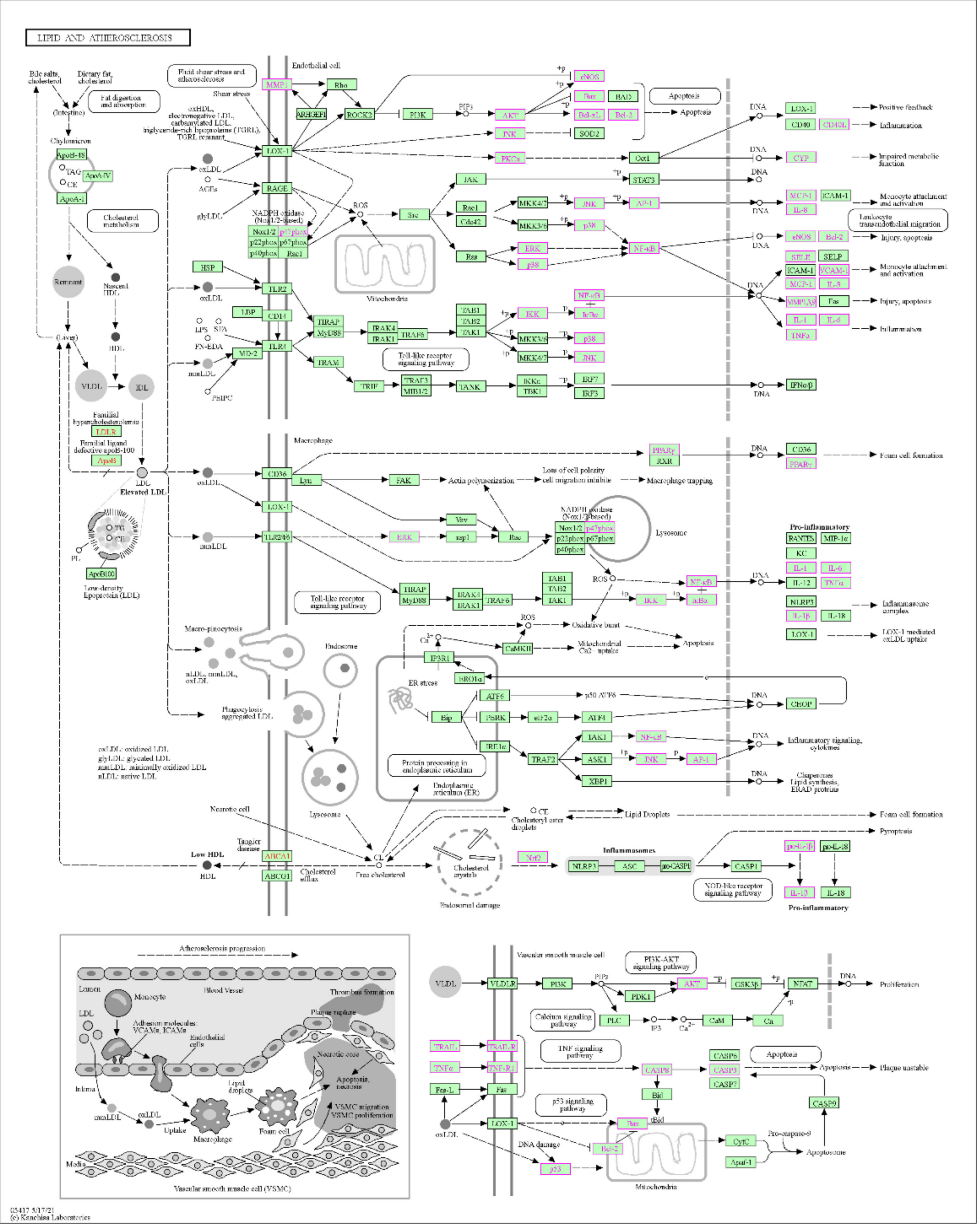
An important pathway of *Ilex kudingcha* treatment of hypertension disease—lipid and atherosclerosis.

KEGG enrichment analysis revealed that the antihypertensive mechanism of *Ilex kudingcha* was mainly concentrated in lipid and atherosclerosis, fluid shear stress and atherosclerosis, the *AGE-RAGE* signaling pathway in diabetic complications, human cytomegalovirus infection, the *TNF* signaling pathway, chemical carcinogenesis receptor activation, hepatitis B, prostate cancer, and Chagas disease (**Figure 7**). The genes associated with the highest number of pathways were *AKT1*, *RELA*, and *MAPK1* (**Table 2**). Lipid and atherosclerosis may be the most important pathway for *Ilex kudingcha* to treat hypertension (**Figure 8**).

**Table 2:** The enrichment pathways corresponding to the intersection genes.

Term	Description	Count	Gene ID
has05417	Lipid and atherosclerosis	31	*AKT1|BAX|BCL2|BCL2L1|CASP3|CASP8|CD40LG|CHUK|CYP1A1|IKBKB|IL1B|IL-6|CXCL8|JUN|MMP1|MMP3|MMP9|NFE2L2|NFKBIA|NOS3|PPARG|PRKCA|MAPK1|RELA|CCL2|SELE|TNF|TNFRSF1A|TP53|VCAM1|NCF1*
hsa05418	Fluid shear stress and atherosclerosis	28	*AKT1|BCL2|CAV1|CHUK|GSTM1|GSTM2|GSTP1|HMOX1|IFNG|IKBKB|IL1A|IL1B|JUN|MMP2|MMP9|NFE2L2|NOS3|PLAT|RELA|CCL2|SELE|TNF|TNFRSF1A|TP53|VCAM1|VEGFA|KEAP1|NCF1*
hashsa05163	Human cytomegalovirus infection	27	*AKT1|BAX|CCND1|CASP3|CASP8|CDKN2A|CHUK|EGFR|IKBKB|IL1B|IL-6|CXCL8|MYC|NFKBIA|PRKACA|PRKCA|PRKCB|MAPK1|PTGS2|RAF1|RB1|RELA|CCL2|TNF|TNFRSF1A|TP53|VhasA*
hsa05207	Chemical carcinogenesis receptor activation	26	*AHR|AKT1|BIRC5|AR|CCND1|BCL2|CYP1A1|CYP1A2|CYP1B1|CYP3A4|EGF|EGFR|GSTM1|GSTM2|JUN|MYC|PGR|PPARA|PRKACA|PRKCA|PRKCB|MAPK1|RAF1|RB1|RELA|VEGFA*

### 3.5 Molecular docking

**Figure 9:**
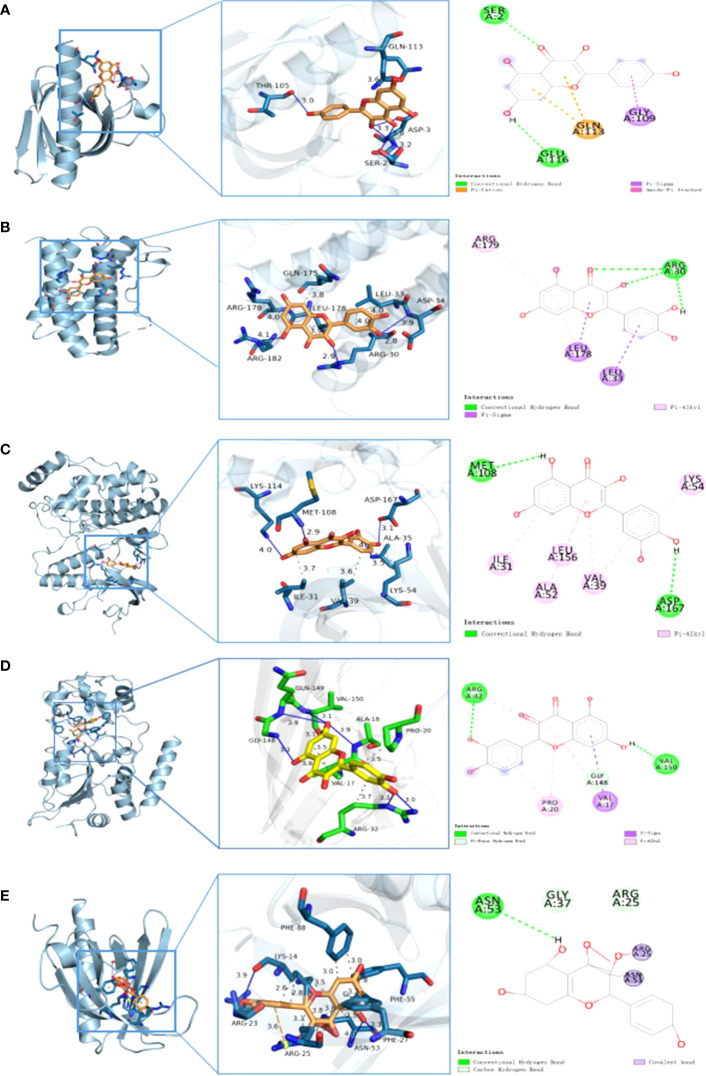
Partial diagram of molecular docking: **(A)** *AKT1*–quercetin, **(B)** *IL-6*–quercetin, **(C)** *MARK1* –quercetin, **(D)** *TNF*– quercetin, and **(E)** *AKT1*–kaempferol.

The structures of the small molecules quercetin (PubChem CID: 5280343) and kaempferol (PubChem CID: 5280863) were downloaded from PubChem. Furthermore, the *AKT1*, *RELA*, *TNF*, *IL-6*, *JUN*, *MAPK1*, *RB1*, and *TP53* proteins with PDB IDs of 2uvm, 1nfi, 5uui, 1alu, 1JUN, 6g54, 1ad6, and 6ggc, respectively, were downloaded from PDB. The binding energies between quercetin and *AKT1*, *RELA*, *TNF*, *IL-6*, *JUN*, *MAPK1*, *RB1*, and *TP53* proteins were −6.1 kcal/mol, −7.4 kcal/mol, −8.8 kcal/mol, −7.0 kcal/mol, −5.7 kcal/mol, −8.4 kcal/mol, −7.4 kcal/mol, and −7.1 kcal/mol, respectively. Furthermore, the binding energies between kaempferol and *AKT1*, *RELA*, and *TNF* proteins were −6.1 kcal/mol, −7.4 kcal/mol, and −8.8 kcal/mol, respectively. The docking results were less than −5 kcal/mol. In general, if the binding energy of the ligand to the target protein is less than −5, the binding between the ligand and receptor protein is stable. **Table 3** displays the detailed results. **Figure 9** comprehensively illustrates several regional molecular docking structures.

**Table 3:** Binding energies of the *Ilex kudingcha* key components to the target gene molecules.

Compounds	Compound target	Docking score	Interaction							
			H-Bond interaction		Hydrophobic interaction		Π-Cation interaction		Π-Π stacking interaction	
			Distance (Å)	Amino acid	Distance (Å)	Amino acid	Distance (Å)	Amino acid	Distance (Å)	Amino acid
Quercetin	Quercetin–*AKT1* complex	−6.1	3.2	SER-2	3.6	GLN-113				
			3.8	SER-2						
			3.3	ASP-3						
			3.0	THR-105						
	Quercetin–*RELA* complex	−7.4	3.0	GLN-29	3.6	GLN-247	2.9	LYS-221		
			3.9	ARG-246	3.9	GLN-247				
			4.0	LYS-218	3.6	LYS-221				
			3.0	GLN-247	3.9	LYS-221				
			2.9	LYS-221	3.9	VAL-244				
	Quercetin–*TNF* complex	−8.8	3.9	ALA-18	3.5	VAL-17				
			3.1	ARG-32	3.8	VAL-17				
			3.2	GLY-148	3.5	PRO-20				
			3.9	GLN-149	3.6	ARG-32				
			3.1	VAL-150						
			3.1	VAL-150						
	Quercetin–*IL-6* complex	−7.0	2.8	ARG-30	4.0	LEU-33				
			3.9	ASP-34	4.0	LEU-33				
			2.9	ARG-30	3.8	GLN-175				
			4.1	ARG-182	3.9	LEU-178				
					4.0	ARG-179				
	Quercetin–*JUN* complex	−5.7	3.0	ARG-302	2.9	ASN-299				
			3.1	ARG-302	3.9	ASN-299				
			3.0	ASN-291	3.7	LEU-294				
			3.2	GLN-290	3.7	LEU-294				
			3.1	SER-292	3.5	ALA-295				
			3.1	ALA-298						
	Quercetin–*MAPK1* complex	−8.4	3.1	ASP-167	3.5	LYS-54				
			4.1	ALA-35	3.6	VAL-39				
			2.9	MET-108	3.7	ILE-31				
			4.0	LYS-114						
	Quercetin–*RB1* complex	−7.4	3.1	SER-391	3.5	TYR-454				
			4.1	SER-391	3.6	ILE-388				
			2.7	GLU-458	2.9	ARG-455				
					3.4	ARG-455				
					4.0	ARG-455				
	Quercetin–*TP53* complex	−7.1	2.8	PRO-219	3.9	GLU-224				
			3.0	ASN-200	3.5	THR-230				
			4.0	HIS-233	3.0	GLU-221				
					4.0	GLU-221				
					3.6	VAL-218				
					3.6	ILE-232				
Kaempferol	Kaempferol–*AKT1* complex	−6.1	3	PHE-88	2.5	ASN-53	3.6	ARG-25	3.2	PHE-27
			2.8	PHE-88	2.6	LYS-14				
			3.3	ASN-53	2.8	LYS-14				
			3.8	ASN-53	3.5	LYS-14				
			4.0	ASN-53	2.5	ARG-25				
			3.9	LYS-14	3.3	ARG-25				
			2	ARG-23	3.8	PHE-27				
			1.8	ARG-23						
	Kaempferol–*RELA* complex	−7.4	2.8	ARG-30	3.5	PHE-187				
			3	ARG-30	3.7	ALA-188				
			2.8	ASN-190	3.8	ASN-155				
			3	PRO-189						
	Kaempferol–*TNF* complex	−8.8	4	PHE-144	3.8	VAL-17				
					3.9	VAL-17				
					3.9	VAL-17				
					3.8	PRO-20				

### 3.6 MD simulation

Molecular docking revealed that quercetin interacts with *TNF* primarily via the formation of hydrophobic forces and hydrogen bond forces. It binds to the amino acid residues VAL-17, PRO- 20, and ARG- 32 of the receptor protein via four hydrophobic bonds and to ALA- 18, ARG- 32, GLY- 148, GLN- 149, VAL- 150, and amino acid residues of the receptor protein via seven hydrogen bonds (**Figure 9D**). To further elucidate the stability of protein–ligand binding, an MD simulation was performed.

#### 3.6.1 RMSD

**Figure 10:**
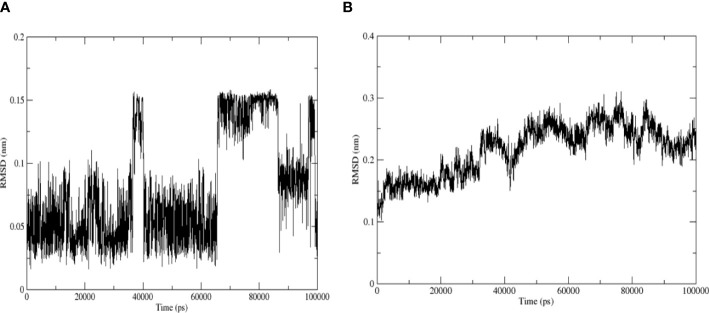
RMSD of quercetin and *TNF* protein 5uui. **(A)** Quercetin; **(B)** *TNF* protein 5uui.

The RMSD curve revealed the RMSD of the structure during the simulation. The movement trajectory of the binding complex demonstrated that the amplitude of quercetin and *TNF* was small during the whole simulation process. Furthermore, the RMSD curve of quercetin revealed that quercetin vibrated near 0–0.15 nm in the whole simulation process and near 0–0.1 nm in the first 30 ns (**Figure 10A**). Moreover, the RMSD curve of TNF demonstrated that the RMSD value kept vibrating at approximately 0.1–0.2 nm at 30 ns, followed by an upward trend of vibration to 0.2–0.3 nm and finally maintaining dynamic stability (**Figure 10B**). Taken together, the results suggest that the complex structure reached a stable conformation after 30 ns.

#### 3.6.2 RMSF

**Figure 11:**
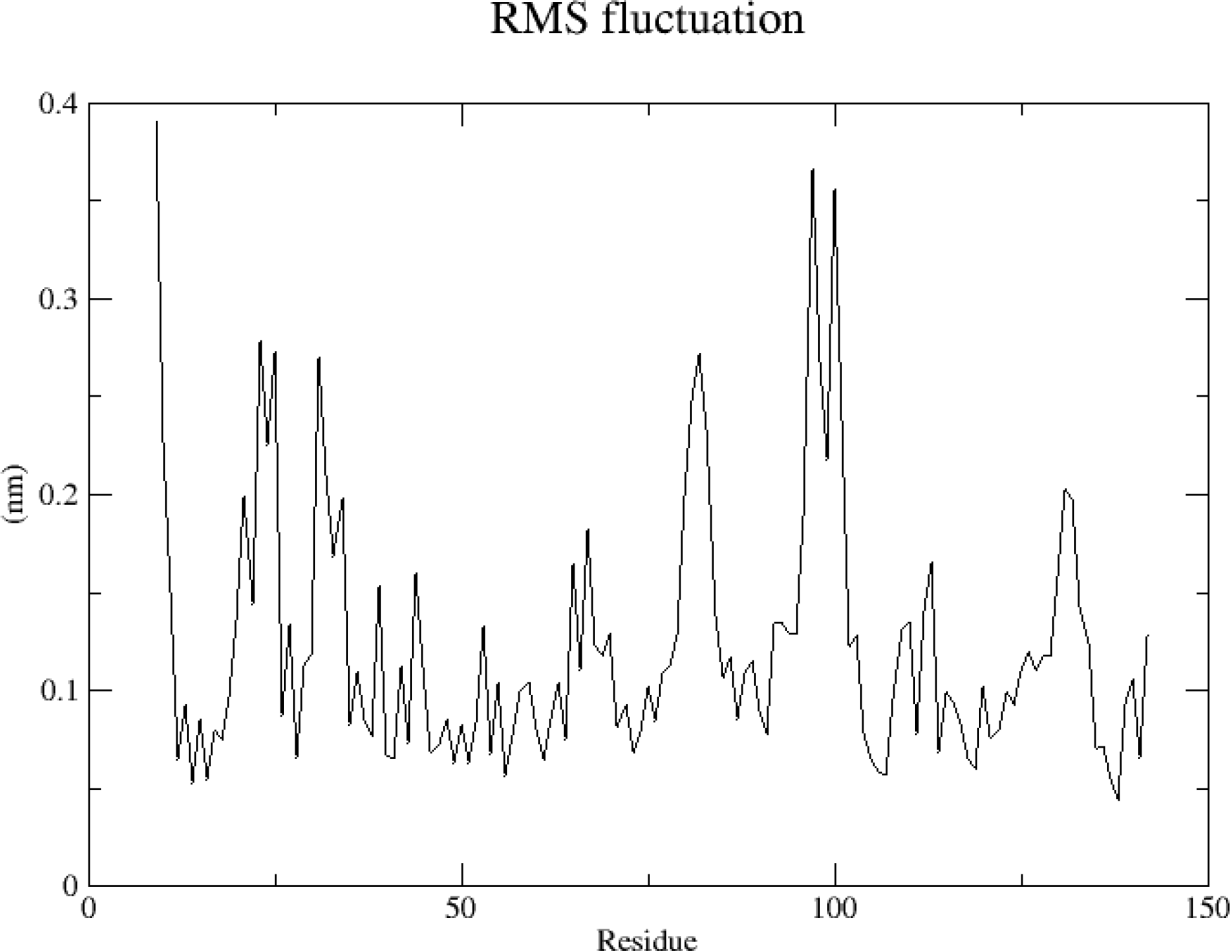
RMSF analysis of *TNF* protein.

RMSF analysis, which indicates the structural fitness of each protein residue, was performed in this study to analyze the flexibility and exercise intensity of amino acid residues in the protein throughout the simulation. After the amino acids of the *TNF* protein 5uui were completely sequenced and the discontinuous part was reordered, RMSF analysis was performed. The *TNF* protein showed weak jitter in simulation, with the amplitude distribution ranging from 0.05 to 0.4 nm; the peak value appeared at Res9, Res97, and Res100; and the RMSF value was the largest. The reason was Res 9 was present at the N-terminal of the protein, and the flexibility of the two ends of the protein was relatively large (**Figure 11**). To summarize, the RMSF value at both ends of the protein was large, whereas that of the amino acid residues was generally small, indicating stable protein–ligand binding.

#### 3.6.3 Rg

**Figure 12:**
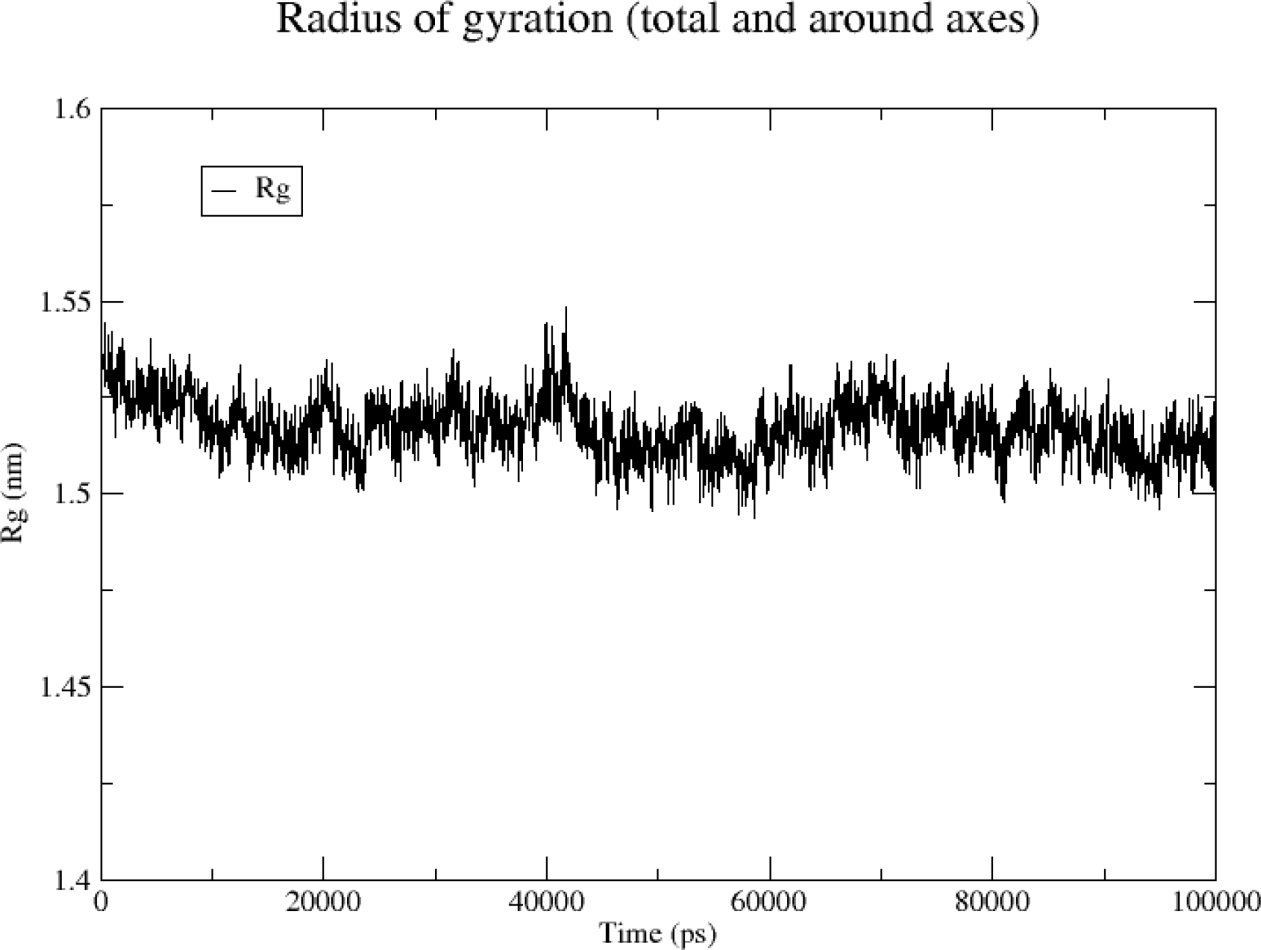
Rg analysis of TNF protein.

Rg can help characterize the compactness of a protein structure and a change in the peptide chain looseness of a protein during simulation. Here, the overall vibration amplitude of the *TNF* protein was small and remained relatively stable at 1.5–1.55 nm (**Figure 12**), indicating that the protein–ligand complex remained relatively stable during simulation.

#### 3.6.4 Hydrogen bonds

**Figure 13:**
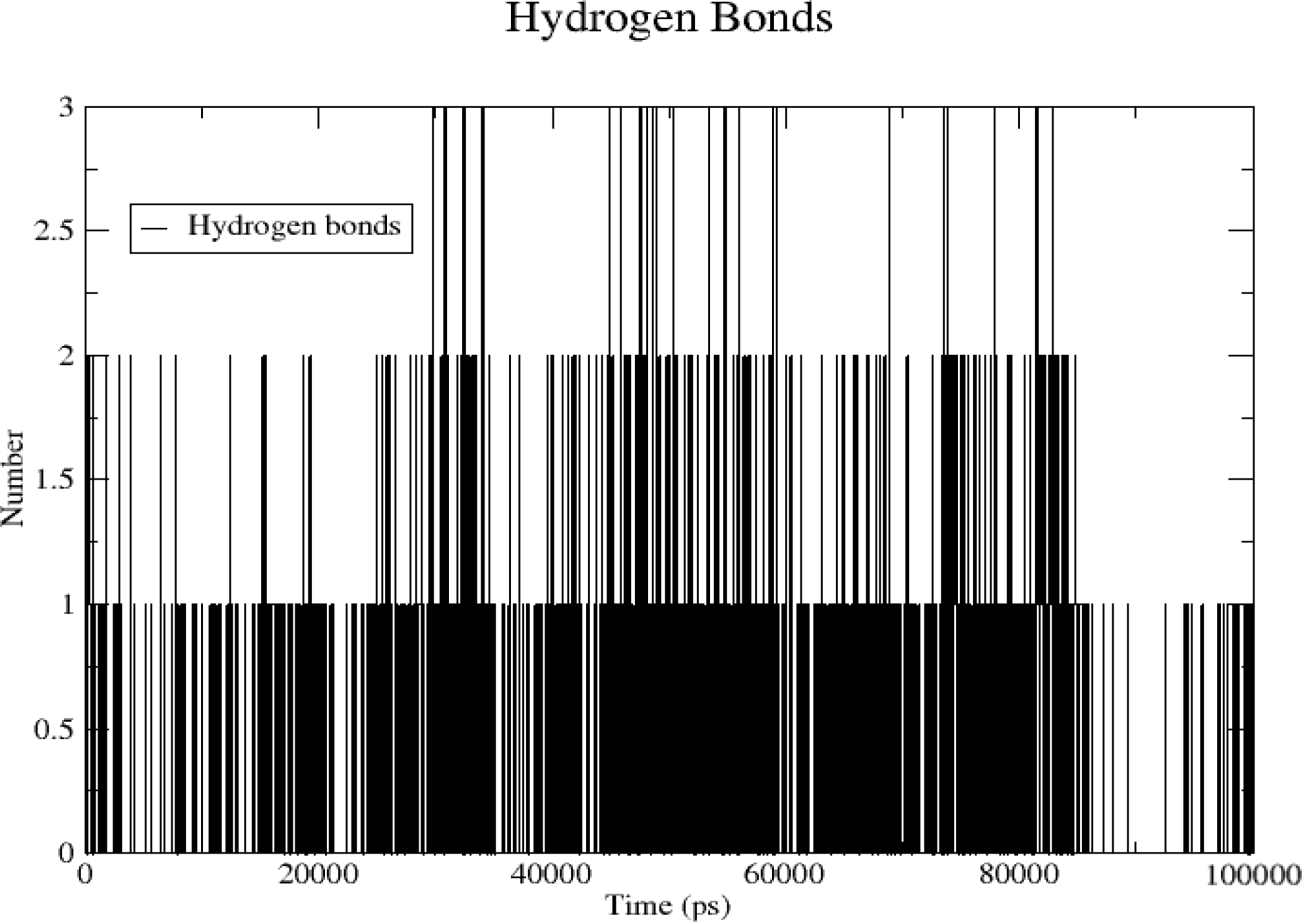
The number of hydrogen bonds with time during 100 ns simulation.

The number of hydrogen bonds formed between the *TNF* protein and quercetin was analyzed with time during a 100-ns simulation. A maximum of three hydrogen bonds were formed at 100 ns, whereas no hydrogen bonds were formed near 90 ns. Mostly, hydrogen bonds were formed 1–2 (**Figure 13**).

## 4 Discussion

TCM is beneficial for treating complicated medical conditions owing to its holistic approach. However, multiple targets, components, and pathways associated with the action mechanism of TCM in disease treatment complicate its development (20). The properties of a Chinese medicinal formula and its mode of action, such as integrity, systematization, and comprehensiveness, are similar to those of network pharmacology (21). The study of the pharmacological mechanisms of Chinese medicines can be well-fitted into pharmacology equations (22).

In the present study, a network pharmacological analysis was performed on the active pharmaceutical components of *Ilex kudingcha* for hypertension. The active compounds quercetin and kaempferol showed the highest number of targets. In the Liu et al. study, the PPI network analysis results showed that the main antihypertensive components of *Ginkgo folium* included kaempferol, quercetin, and luteolin (23). Ye et al. found that the antihypertensive active ingredients of *Eucommia ulmoides* included quercetin, kaempferol, and rutin (24). Yang et al. reviewed Chinese herbal prescriptions for hypertension and found that components including quercetin, luteolin, linolenin, and kaempferol were closely associated with the regulatory targets of hypertension (25). Moreover, the present molecular docking results showed that the active components exhibited effective binding ability with most of the target genes. Quercetin is a typical natural flavonoid, which is a secondary metabolite found in plants and is thought to be one of the bioactive substances found in fruits and vegetables; it is beneficial in maintaining cardiovascular health (26). Quercetin is advantageous in decreasing blood pressure and inflammation at the highest effective dosage of 500 mg of the aglycone form (27). Kaempferol, another natural flavonoid, is an effective anti-inflammatory, antioxidant, and anticancer agent and is documented in treating several illnesses, including diabetes, obesity, and cancer (28). Quercetin and kaempferol exert the most significant hypolipidemic effects at a low concentration of 15 μM (29). Kaempferol exerts its effect in various ways, including decreasing the activity of human T cells and via phosphoinositide-3-kinase (*PI3K*)/*AKT* via human T-lymphotropic virus type 1 signaling pathways. The leukemia/lymphoma virus inhibits the production of several proteins that are the hallmarks of epithelial–mesenchymal transition, such as Slug, N-cadherin, E-cadherin, and Snail and indicators of metastasis, including matrix metallopeptidase 2 (28).

The core genes were obtained based on the PPI network, and the main targets were *RELA*, *AKT1*, *JUN*, *TP53*, *TNF*, *MAPK1*, *IL-6*, *RB1*, *CAV1*, and *EGFR*, and their degree values were 58, 58, 58, 56, 50, 50, 44, 32, 32, and 32, respectively. *AKT1* is associated with hypertension, and *AKT1* mutations greatly increase the risk of hypertension. Furthermore, due to damage to *AKT1* signaling pathways, insulin cannot be effectively transported to the vascular system by the action of endothelial nitric oxide synthase (*eNOS*), leading to obesity and insulin resistance, followed by artery dysfunction and hypertension (30, 31). *TNF* and *IL-6* are two proinflammatory cytokines associated with atherosclerosis, plaque development, and increased cardiovascular risk (32–34). In the present study, the core targets were involved in abnormal biological processes including inflammatory responses, lipid metabolism, vascular endothelial functions, and energy metabolism, which lead to hypertension.

GO enrichment was performed to identify disease–drug intersection genes. The findings revealed that the biological effects of the active ingredients of *Ilex kudingcha* were exerted via signaling cytokine receptor binding, cytokine activity, receptor–activator activity, and receptor–ligand activity binding of transcription factors, which may have affected membrane rafts, membrane microdomains, cytoplasmic vesicle lumens, secretory granule lumens, plasma membrane rafts, and vesicle lumens, thus contributing to cellular responses to oxidative stress, epithelial cell proliferation, and biological processes including wound healing, reaction to xenobiotic stimulation, and chemical stress. The KEGG enrichment analysis indicated that the action mechanism of *Ilex kudingcha* in non-hypertensive atherosclerosis was primarily associated with lipids, fluid shear stress, and human cytomegalovirus infection, the *AGE–RAGE* signaling pathway in diabetes complications, and *TNF* activation of the chemical carcinogenesis signaling pathway. The lipid and atherosclerosis map (**Figure 8**) showed that the active ingredients of *Ilex kudingcha* acted on many targets and formed an interactive relationship with atherosclerosis, fluid shear stress, and *MAPK*, *TNF*, and *PI3K/AKT* signaling pathways, all of which helped regulate oxidative stress and vascular hardness. *TNF* inhibits inflammatory responses while reducing blood pressure and excreting salt through the kidneys (35). The *PI3K*/*AKT* signaling pathway controls the cytoskeletal rearrangement and phenotypic transformation of arterial smooth muscle cells, affects the excitability of sympathetic nerves and the function of vascular endothelial cells, and antagonizes angiotensin II (36, 37). Dyslipidemia and hypertension are considerable risk factors associated with the development of atherosclerotic cardiovascular illnesses (38) and induce various vascular events by promoting atherosclerosis occurrence and development as well as plaque formation (39–41). The pathophysiological interaction between hypertension and dyslipidemia, which includes oxidative stress, proinflammatory activities, renin–angiotensin–aldosterone system activation, and endothelial dysfunction, has been supported by accumulating evidence (42). A considerable positive effect of lipid-lowering therapy has been observed in older patients with obesity and hypertension; the therapy markedly lowers obesity-related indicators including blood pressure, lipid levels, and glucose levels as well as considerably improves atherosclerosis-related symptoms without side effects (43). Several *in- vivo* experiments have shown that the *Ilex kudingcha* extract reduces atherosclerosis in apoE-deficient mice by decreasing cholesterol buildup in macrophages (44). Considering their effects on hemorheological properties, the total saponins in *Ilex kudingcha* may possess considerable therapeutic value for treating hypercholesterolemia and atherosclerosis (45). *Ilex kudingcha* exerts potent anti-inflammatory effects on LPS-induced inflammatory responses by inhibiting *NF-κB* and *MAPK* pathways (10). *Dioscorea opposita* Thunb., a common staple food in China, exerts antihypertensive effects by inhibiting the endothelin-converting enzyme and antioxidant activity in 2K1C hypertensive rats (46). Furthermore, the binding activity of the protein and its ligand was explained by molecular docking in the present study. The results showed that quercetin and kaempferol bound well to the corresponding target proteins. Small molecules interacted with proteins mainly by forming hydrophobic and hydrogen bonds. The lowest binding energy of *TNF* with quercetin and kaempferol was −8.8 kcal/mol, followed by −8.4 kcal/mol of *MARKI* with quercetin. The docking result was less than −7.0 kcal/mol, indicating that the ligand and receptor exhibited strong binding activity (47). The MD simulation analysis results confirmed the stable binding of *TNF* and quercetin. MD simulation has been widely used in the biomedical field to study conformational transformations caused by protein mutations or ligand binding/debinding. It provides some findings that are difficult to obtain in traditional biochemical or pathological experiments, such as the detailed effects of mutations on protein structures and protein–protein/ligand interactions at the atomic level (48). MD simulation can provide valuable information in deciphering the functional mechanisms of several biomolecules including proteins/peptides, overcoming existing sampling limitations in docking analysis (49).

## 5 Conclusion

*Ilex kudingcha* can play a role in lowering blood pressure via multiple components, targets, and pathways. This study provides insights into how the hypolipidemia-, antioxidation-, and atherosclerosis-related gene inhibitory effects of *Ilex kudingcha* are associated with its mode of action in treating hypertension. The action targets include *RELA*, *AKT1*, *TNF*, *IL-6*, and *MARK1*. Studies that are pertinent to these findings rely on data from already-existing databases and lack experimental validation. Therefore, further studies are required to verify the accuracy of the present findings *in vitro* and *in vivo*.

## Data availability statement

The original contributions presented in the study are included in the article/supplementary material. Further inquiries can be directed to the corresponding authors.

## Author contributions

FL wrote and revised the manuscript and constructed the tables and figures. MY and RH revised the manuscript. YQ and YH conceived the study and revised the manuscript. All authors have read and approved the final manuscript. Data authentication is not applicable.
